# A Study on the Oxidative Functionalization of a Poplar Biochar

**DOI:** 10.3390/molecules30051048

**Published:** 2025-02-25

**Authors:** Antonella Di Vincenzo, Ettore Madonia, Calogero Librici, Paola Bambina, Delia Chillura Martino, Susanna Guernelli, Paolo Lo Meo, Pellegrino Conte

**Affiliations:** 1 Department of Biological, Chemical and Pharmaceutical Sciences and Technologies, University of Palermo, V. le delle Scienze ed. 17 S. Cannizzaro, 90128 Palermo, Italy; antonella.divincenzo@unipa.it (A.D.V.); delia.chilluramartino@unipa.it (D.C.M.); 2 Department of Agricultural, Food and Forest Sciences, University of Palermo, V. le delle Scienze ed. 4, 90128 Palermo, Italy; ettore.madonia@unipa.it (E.M.); calogero.librici@unipa.it (C.L.); paola.bambina@unipa.it (P.B.); pellegrino.conte@unipa.it (P.C.); 3 Department of Chemistry “G. Ciamician”, University of Bologna, Via P. Gobetti, 83, 40129 Bologna, Italy; susanna.guernelli@unibo.it

**Keywords:** biochar, oxidation, sulfonitric mixture, piranha mixture, FFC-NMR relaxometry, adsorption

## Abstract

This study investigates the functionalization of a poplar biochar (PB), obtained by high-temperature pyrolysis, under oxidative conditions typically used in organic synthesis. In particular, concentrated nitric acid, a sulfonitric mixture and a piranha mixture were applied as oxidants at different temperatures and reaction times. In order to assess the outcome of the reaction conditions on the characteristics of the resultant products, these were characterized by a combination of imaging (SEM), spectroscopic (ATR-FTIR, RAMAN) and FFC-NMR relaxometric techniques. The latter techniques, rationalized in terms of the Kohlrausch-type stretched exponential kinetic model, were analyzed using a recently developed heuristic Monte Carlo method, providing insights into the water dynamics within material pore networks. Additionally, the water-holding capacity of the modified biochars and their abilities to adsorb some model dyes were evaluated. The results clarify the relationship between oxidative treatment conditions and biochar properties, highlighting their impact on both structural modifications and water dynamics within the porous network, and enabling us to identify the best reaction conditions for optimizing the features of the oxidized product.

## 1. Introduction

The treatment and reuse of wastewater are increasingly vital in addressing environmental protection, resource remediation, and the sustainable management of natural resources [1,2,3]. Particular attention is presently directed towards the removal of emerging pollutants such as dyes and pharmaceuticals [3,4,5,6,7], and heavy metals [8] as well. Among the various methods available, adsorption using activated carbonaceous materials stands out for its simplicity and efficiency [9]. Biochars, in this regard, have emerged as promising sorbents due to their low cost, wide availability and the fact that they represent the potential to utilize underexploited biomass, aligning with the principles of the circular economy and resource efficiency [10,11,12]. According to the European Biochar Certificate (EBC), biochar is “*a porous, carbonaceous material that is produced by pyrolysis of biomass and is applied in such a way that the contained carbon remains stored as a long-term C sink or replaces fossil carbon in industrial manufacturing. It is not made to be burnt for energy generation*” [13]. Due to its peculiar porous structure and high specific surface area, biochar can effectively function as a soil conditioner, enhancing fertility and water-holding capacity [14]. The properties of a biochar are largely variable, depending on the nature of the starting biomass and the operative conditions employed for its preparation. In particular, features such as texture properties, pH, surface charge and functional groups, as well as the ability to interact and exchange chemicals and soil nutrients with aqueous media [15,16,17], can be critically affected.

Chemical modification of biochars has been shown to be an interesting strategy for modulating and, possibly, improving their characteristics [12,18]. For this purpose, particular attention has been paid to oxidative treatment with various oxidizing systems, under both acidic and alkaline conditions [19]. Hence, lots of different protocols have been formulated to accomplish the task, allowing for the use of H_2_O_2_, HNO_3_ or HNO_3_/H_2_SO_4_ (sulfonitric mixture) as the sacrificial oxidant [20,21,22]. The outcome of using these treatments on biochars of different origins has been the object of various studies, particularly those aiming to understand the consequent increase in the amount of oxygenated functional groups present [18], as well as those aiming to obtain information useful for understanding the possible mechanisms of the slow degradation of biochar occurring after its application in soil conditioning [23,24]. Of course, a main problem in these studies is the extreme variety in the origins of the starting biomass used for biochar production. Regardless, oxidative chemical modification often results in a large improvement in the abilities of a material to adsorb both organic and inorganic pollutant species. Just to cite a few recent examples, the oxidative treatment of a weed-based material with HNO_3_ improved the adsorption of methylene blue [22]. Other materials derived from maple [25] and straw [26] showed increased sequestration abilities for Pb^2+^ and Cd^2+^ ions, respectively. A microalgal biochar treated with a sulfonitric mixture was successfully employed for the adsorption of gaseous H_2_S [27]. In addition, oxidized biochars have been used to improve soil buffer capacity [28].

An important point to address, as far as the possible chemical modification of a biochar material is concerned, is how the oxidizing treatment can affect the mobility of an aqueous medium within its meso- and microporous network [14]. This, in turn, leads to the consequent ability of the material to exchange matter and nutrients, after its application for soil conditioning, and to sequester potential pollutant species as well. Therefore, as a further development of our previous works [15,18,29,30], in the present study, we examined the oxidation of a poplar biochar (PB) obtained at a high pyrolysis temperature (1200 °C) with three different common oxidation systems. In particular, the “piranha mixture” (H_2_O_2_ and conc. H_2_SO_4_ mixture), conc. HNO_3_ and the “sulfonitric mixture” (HNO_3_ and H_2_SO_4_ mixture) were chosen, varying the temperature and the reaction time. Furthermore, in order to evaluate how the different oxidation methods cause physicochemical and structural modifications to the products obtained, we characterized the materials by combined imaging (SEM), spectroscopic (ATR-FTIR) and relaxometric (FFC-NMR) techniques. Finally, we evaluated the abilities of the obtained materials to adsorb some organic dyes as pollutant models.

## 2. Results and Discussion

### 2.1. Preparation of Modified Biochars

As highlighted above, the chemical functionalization of biochars can be accomplished by various oxidative protocols. In this study, we took into consideration the use of acidic oxidizing systems such as the piranha (conc. H_2_SO_4_ and 30% aq. H_2_O_2_ 3:1 *v*/*v*) and sulfonitric (conc. H_2_SO_4_ and 68% aq. HNO_3_ 3:1 *v*/*v*) mixtures, or even simple concentrated nitric acid. Notably, the latter two systems might also decorate the biochar aromatic scaffold with nitro groups, liable to further chemical transformation (such as reduction to –NH_2_, for instance, via established protocols [31]). It is important to stress that the operational conditions have a significant influence on the reaction course, in terms of final yields and extent of functionalization. Reactions were carried out at various temperatures (0 °C, 27 °C, 60 °C, and 90 °C) and reaction times (10, 30, 60, 90, and 180 min), with 60 °C for 30 min serving as the standard condition. The reaction conditions used and the corresponding yields are summarized in Table 1. It is interesting to observe that the apparent weight yields (calculated as the ratio between the mass of the isolated product and the starting material) are consistently below 100%. Mass losses may be attributed to both the significant erosion of the carbonaceous substrate due to the relatively severe oxidation conditions and the elution of soluble low-molecular-weight species. The latter ones may derive, in turn, from the thermal decomposition of lignin or other metabolites (oligosaccharides, flavonoids, polyphenols) already present in the original biomass. This hypothesis is supported by the characteristic brown color the former washings show during the work-up procedure. This significant mass loss is noteworthy. For instance, Hummers-like oxidation of graphene to graphene oxide indeed results in a weight increase due to the incorporation of oxygen atoms, which leads to the formation of phenolic and carboxylic functional groups, whose presence can be evidenced and quantified by trivial titration with a standard base [32]. The latter consideration suggested the opportunity to carry out, by analogy with previous work [32], an analytical characterization via acid–base titration. Interestingly, the pristine BP exhibited a slight alkalinity (8 ± 1 μeq g^−1^), likely due to trace alkaline salts (e.g., carbonates) retained during processing. Titration analysis of the materials B1–B7 obtained with the sulfonitric mixture evidenced the presence of acidic functional groups (both carboxyl and phenol, presumably, with apparent p*K*_a_ values spanning from 3.5 to 9.8), whose amount per g of material regularly increases on increasing the reaction time or temperature. A comparable amount of acidic groups was found also for two representative materials (A3, A5) obtained with the piranha mixture.

### 2.2. SEM Micrography

**Figure 1:**
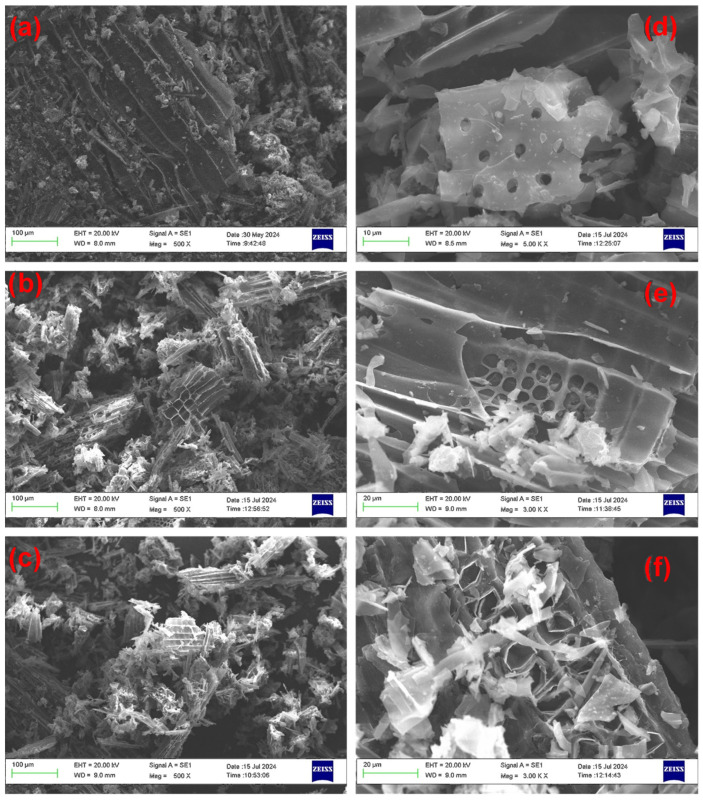
Selected SEM micrographs for materials PB (**a**), A5 (**b**), B7 (**c**,**f**), B2 (**d**), and B4 (**e**).

Morphological characterization of the samples provides crucial insights into the effects of the oxidative treatments on the pristine biochar. Specifically, it allows us to assess n.d. the extent of erosion and mechanical stress the material undergoes under varying reaction conditions. Representative SEM micrographs are presented in Figure 1 (the complete collection is reported in the Supporting Information). The starting product PB shows a fair preservation of the biomass cellular structure (Figure 1a), with distinct and ordered mesoporous substructures clearly visible. As the oxidation conditions become harsher and harsher, the progressive erosion of the material structure becomes apparent. For instance, the images for samples A5 and B7 (Figure 1b and Figure 1c, respectively), obtained by reaction at 90 °C, show significant damage. The originally well-defined cell structure deteriorates into a disordered and fragmented state, highlighting the impact of elevated temperature on structural integrity. The effect of rising temperature is particularly evident in the case of the sulfonitric mixture (samples B2, B4 and B7, Figure 1d, Figure 1e and Figure 1f, respectively). At a relatively mild temperature of 27 °C (B2, Figure 1d), the micrographs show pore structures that closely resemble those of the pristine biochar. However, increasing the temperature to 60 °C (B4, Figure 1e) leads to substantial erosion of the mesoporous substructures, which ultimately undergo extensive collapse at 90 °C (B7, Figure 1f). Overall, these observations support the hypothesis that oxidative treatments result in significant erosion of the biochar matrix.

### 2.3. ATR-FTIR and Raman Spectroscopy

**Figure 2:**
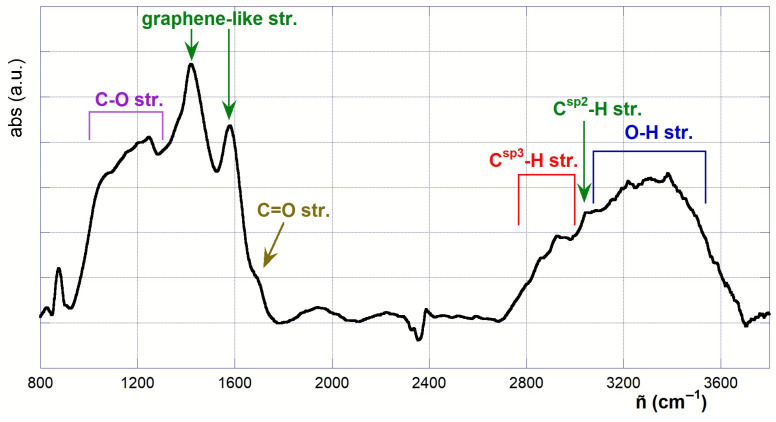
ATR-FTIR spectrum of PB.

In order to assess the outcome of the oxidizing treatment on the chemical structure of the materials, ATR-FTIR spectroscopy analyses were performed. The complete spectrum of the pristine material PB (depicted in Figure 2) features two main adsorption regions, located in the 3700~2700 and 1800~800 cm^−1^ wavenumber ranges. In detail, signals present in the first region can be attributed to the O-H and C-H stretching modes of the hydroxyl groups and aliphatic moieties present in the material, and to the adsorbed water molecules possibly present. An analysis of the second derivative of the observed band system suggests the superimposition of not less than twenty different contributions, the most prominent of which are located at 3587, 3497, 3385, 3300, 3222, 3145 (O-H str.), 3044 (C*^sp2^*-H str.), 2920, 2852 and 2790 (C*^sp3^*-H str.) cm^−1^. Similarly, the second region also features a plethora of superimposed bands, among which it is easy to identify at least two main signals centered at 1580 and 1422 cm^−1^, accounting for the presence of an extended C*^sp2^* graphene-like carbonaceous scaffold. The concomitant broad absorptions in the region at ca. 1300~950 cm^−1^, in turn, are consistent with the stretching of C-O bonds, accounting for the presence of oxygenated functions (-OH, -OR). In addition, a tiny shoulder at ca. 1711 cm^−1^ indicates the presence of carbonyl groups. Notably, since this spectral region is less affected by the possible presence of adsorbed water, its analysis is much more informative for assessing the outcome of the oxidizing treatment.

**Figure 3:**
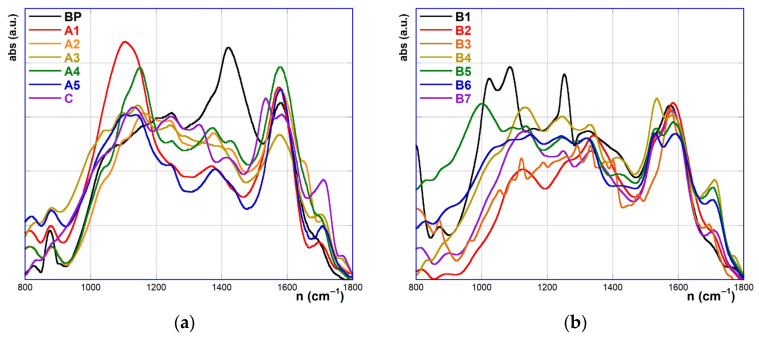
Superimposed ATR-FTIR spectra of the materials in the 1800–800 cm^−1^ region: (**a**) PB, A1–A7, C; (**b**) B1–B7.

The superimposed spectra of the oxidized biochars (limited to the 1800~800 cm^−1^ region) are shown in Figure 3. The most noticeable change is the decrease or near disappearance of the signal at 1422 cm⁻^1^, reflecting the loss of C=C bonds and the introduction of new functionalities. At the same time, deep and very complex changes in the C-O stretching region can be envisaged, generally resulting in the intensity increase of the signals, or even in the appearance of new bands. Of course, these changes can be easily explained by assuming that oxidation involves the introduction of new oxygenated functionalities on the carbonaceous scaffold, with an increase in the fraction of *sp^3^*-hybridized C atoms.

Moreover, in most cases, oxidative treatments also cause the appearance of a new signal centered at ca. 1770 cm^−1^ (C=O carbonyl stretching), consistent with the presence of aryl ester groups or lactone rings. The latter finding may be justified, from a mechanistic standpoint, assuming the occurrence of a Bayer–Villiger-like process on some ketone functional groups, either already present in PB or possibly formed during the former steps of the oxidation process. Additionally, in the case of the sulfonitric mixture or the nitric acid oxidation, further signals centered at ca. 1530 and 1330 cm^−1^ can be identified, which account for the possible presence of –NO_2_ groups decorating the carbonaceous scaffold. The latter ones are likely introduced via a trivial electrophilic substitution (*S_E_Ar*) reaction.

A closer inspection of the spectra enables us to consider the effects of the reaction time and temperature in more detail. Considering the appearance of the 1770 cm^−1^ C=O band, spectra comparison suggests that an operational temperature of 60 °C affords the most effective functionalization both with the piranha mixture (samples A1, A2, A3, A5) and the sulfonitric mixture (samples B1, B2, B4 and B7). Similarly, a reaction time of 30 min appears as the best choice (samples B3–B6 and A3–A4). Interestingly, the signal at 1530 cm⁻^1^ (indicative of NO_2_ groups) follows the same trends as the carbonyl band, confirming that both temperature and time critically influence the extent of oxidation. Furthermore, in the 1250–850 cm⁻^1^ region, significant spectral changes also occur, reflecting an increase in oxygenated functionalities. For the sulfonitric mixture, signal intensities follow a bell-shaped trend as the reaction time increases, peaking at 30 min (~1130 cm⁻^1^). In contrast, the effect of temperature is more nuanced: strong absorption bands at 1022 cm⁻^1^ and 1106 cm⁻^1^ are observed for B1 (0 °C), but these bands almost disappear at higher temperatures. A similar trend is observed with the piranha mixture, where a peak at 1105 cm⁻^1^ in sample A1 (0 °C) disappears under harsher conditions. Conversely, the effect of the reaction temperature is more articulated. Two strong absorption bands appear at 1022 and 1106 cm^−1^ for the product B1 obtained at 0 °C, which almost disappear at higher reaction temperatures. Something similar occurs also for the reaction with the piranha mixture, with the appearance of a strong signal at 1105 cm^−1^ for product A1, which almost disappears for the other samples, obtained at higher temperatures. These observations suggest that the oxidation process first causes an oxygen transfer onto some C*^sp2^*=C*^sp2^* double bond, with the formation of epoxide or *gem*-diol functionalities (and consequent change in hybridization for the relevant C atoms, as accounted for by the sudden disappearance of the 1422 cm^−1^ band). These functionalities, however, easily undergo further oxidation to some carbonyl-containing group (ketone, carboxylic acid, ester, lactone).

**Figure 4:**
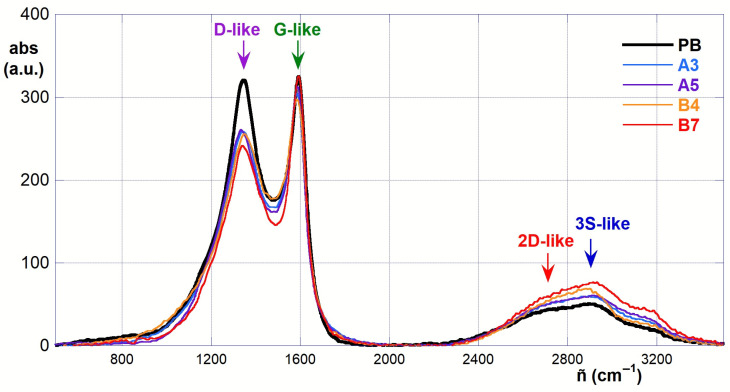
Superimposed Raman spectra of PB, A3, A5, B4 and B7.

In order to gain further complementary insights, Raman spectra of some selected representative samples were acquired. In particular, pristine PB and materials A3, A5, B4 and B7 were considered for evaluating the outcome of the two main oxidizing systems at both the “best” (60 °C, 30 min) and the harshest (90 °C, 30 min) conditions. The obtained spectra (after suitable baseline correction and normalization) are depicted in Figure 4 and feature the typical graphene-like bands centered at ca. 1343 (D-like) and 1589 (G-like) cm^−1^ (with minor differences from sample to sample), together with a system of broad superimposed bands in the 2400–3400 cm^−1^ region (analogous to the 2D and 3S graphene bands system) [32]. Compared to graphene materials, the former two bands appear broader and much more overlapped. This can be attributed to the quite disordered amorphous microscopic structure typical of biochars. Therefore, the intensity ratio *I*_D_/*I*_G_ between the two bands, which is usually exploited to evaluate the amounts of defects in the graphene sheet C*^sp2^* backbone, must be considered with care and may just provide a semi-quantitative evaluation of how the C*^sp3^*/C*^sp2^* ratio changes due to the oxidizing treatment of the pristine biochar material. Similar care, of course, is needed in evaluating the ratios *I*_2D_/*I*_G_ and *I*_2D_/*I*_3S_, which are related to the average number of sheets and the extent of the graphene-like microdomains. The relative intensities of the signals can be retrieved by suitable deconvolution analysis of the spectra as a sum of Voigt functions [33].

Apparently, the oxidizing treatment of the pristine PB causes a decrease in the D-like band and an increase in the intensity of the 2D/3S-like system. However, analysis of the intensity ratios, obtained from the deconvolution analysis, tells a more articulated story. In fact, significant variations occur also in the bandwidths. The apparent *I*_D_/*I*_G_ ratio passes from 1.75 for PB to 2.33 and 1.11 for A3 and A5, respectively, and to 2.07 and 1.35 for B4 and B7, respectively. These findings suggest that, with both the piranha and sulfonitric mixtures, under the best reaction conditions, the relative amount of *sp3*-hybridized C atoms increases due to the increased amount of functional groups introduced. However, over-oxidation of the materials under the harshest conditions also induces a sort of “chemical collapse” of the microscopic structure, with a consequent increase in the fraction of unsaturated carbons. Deconvolution analysis confirms the progressive increase in the intensity of the 2400–3400 cm^−1^ band system upon an increase in the reaction temperature with both the oxidizing systems (the cumulative signal intensity ratios pass from 0.23 for PB to 0.29 and 0.35 for A3 and A5, respectively, and to 0.33 and 0.44 for B4 and B7, respectively). The *I*_2D_/*I*_3S_ ratios also show intriguing variations. Starting from the value of 2.09 for PB, values pass to 2.44 and 3.83 for A3 and A5, respectively, and to 0.75 and 1.22 for B4 and B7, respectively. Again, these results suggest that more extensive graphene-like microdomains occur in the oxidized materials upon increasing the reaction temperature. However, a clear difference occurs between the two different oxidizing systems, with the piranha mixture causing the formation of larger microdomains.

### 2.4. Water-Holding Capacity and Porosimetry

The water-holding capacity (WHC), defined as the amount of water retained per unit weight of material after equilibration at room temperature with an excess of water, is a critical parameter for characterizing biochars [34]. In fact, it plays a crucial role in the effectiveness of biochar as a soil conditioner, as it directly influences the material’s ability to exchange water and nutrients with the soil. Hence, we can assume that maximizing WHC is a reasonable criterion for individuating the most suitable oxidative treatment conditions. The WHC values obtained for the materials (Table 2) enable us to make a further assessment of the effect of the type of oxidation treatment and the experimental conditions on the product obtained. In all cases, a significant increase in WHC values can be observed compared to pristine PB, indicating an enhancement in the hydrophilic character of the materials, due to the introduction of new chemical functionalities. Nevertheless, caution is needed in closely analyzing the results, because in principle, the simultaneous occurrence of extensive oxidative erosion may also lead to the collapse of the porous microstructure of the material [14]. The latter circumstance, in turn, would cause a decrease in the WHC values.

A close inspection of the results reported reveals some interesting trends. Considering the effect of the increase in the reaction temperature, with the piranha mixture, the WHC value stays roughly constant up to 60 °C and then rises for the sample A5 obtained at 90 °C. Conversely, with the sulfonitric mixture, WHC values show a bell-shaped trend, increasing up to 60 °C, and then dropping down at 90 °C. These findings suggest that the sulfonitric mixture overall provides harsher reaction conditions for PB oxidation than the piranha mixture, causing the aforementioned partial collapse of the microporous structure due to over-oxidation at lower temperatures. This idea is confirmed by the trends as a function of the reaction time. In fact, with the sulfonitric mixture, WHC values decrease as the reaction time passes from 10 min up to 60 min, and they rise again with a 90 min reaction time. Therefore, overall, these findings suggest that the oxidation process proceeds via a complex pathway. Under milder conditions, oxidation primarily causes superficial decoration of the carbonaceous material with oxygenated functional groups, enhancing hydrophilicity while preserving the porous structure. However, under harsher conditions (higher temperature or extended time), the oxidation progresses to deeper structural erosion, causing a partial collapse of the microporous network. In some cases, this extensive oxidation may lead to a secondary increase in WHC due to the creation of smaller pores or increased surface hydrophilic character.

Finally, complementary insights were achieved by porosimetric analysis of four selected materials, namely PB, A3, A5 and B7, using the N_2_-sorption BET and BJH analysis methods. The pristine PB shows a specific surface area *S* as large as 194.4 m^2^g^−1^ (BET), a specific pore volume *V* of 98 mm^3^g^−1^, and an average pore diameter *D* of 19.2 Å (BJH). The oxidative treatment apparently causes a dramatic collapse of the structure. The apparent *S* values fall down to 7.9, 7.6 and 4.0 m^2^g^−1^ for A3, A5 and B7, respectively. Similarly, *V* values of 14, 13 and 9 mm^3^g^−1^, respectively, are found. Strangely, however, no significant variations are found for *D* values. Although, from a quantitative standpoint, the reliability of these few results may appear questionable, they indeed provide convincing support to the idea that an extensive collapse of the mesoporous structures of the original biomass occurs due to the oxidizing treatment, as evidenced by SEM micrography.

### 2.5. FFC-NMR Relaxometry

The characterization of the materials was completed by means of NMR relaxometry. In particular, the Fast Field Cycling (FFC-NMR) technique provides a powerful tool for assessing the texture properties and the functional mobility of aqueous media within both organic and inorganic meso- and microporous materials [35,36,37], such as soils, zeolites, nanosponges [38], and biochars [39] too. The principles and applications of the FFC technique have been described in detail in the literature [40,41,42,43]. In brief, the porous material is saturated with water. Then, by applying a suitable sequence of magnetic fields and pulses, it is possible to acquire the spin-lattice relaxation kinetics profiles at different magnetic fields, routinely covering a ^1^H Larmor frequency range (ν_L_) spanning from 10 kHz to 10 MHz. Hence, the relevant longitudinal relaxation rates (*R*_1_), and consequently the Log (*R*_1_) vs. Log (ν_L_) NMRD dispersion curves can be easily retrieved. An analysis of these curves enables us to obtain the correlation times *τ*_c_ relevant to the different dynamic regimes occurring in the system. In particular, according to the well-known Bloembergen–Purcell–Pound (BPP) theory, *τ*_c_ can be considered as the average time needed for a water molecule to rotate one radian, or to translate for a distance as large as its gyration radius [44,45]. In general, for the chemically modified biochars, the relaxation kinetics profiles deviate from a simple exponential trend, commonly observed for homogeneous systems, but should rather be adapted using the well-known Kohlrausch-type “stretched exponential” model Equation (1) [46,47]:

*M* (t) = *M*_∞_ + (*M*_0_ − *M*_∞_)·*exp*[−(*R*_1_·t)*^n^*] (1)

where *M* (t) is the time-dependent magnetization of the sample, *M*_0_ and *M*_∞_ are the starting and equilibrium magnetizations, respectively, and *n* is the exponential stretch parameter. This peculiar finding is consistent with the occurrence of some inhomogeneity in the behavior of the water molecules within the pore network. This can be easily justified, in turn, considering the different solvation requirements of the pore surface. In fact, the water molecules interacting with the more hydrophilic chemically modified portions of the biochar surface experience a different molecular mobility, and consequently a different relaxation behavior, in comparison to the molecules interacting with the more hydrophobic non-modified portions. Notably, for any biochar sample, only fair or even no dependence can be envisaged between *n* values and the operational strength of the relaxation magnetic field. For the pristine PB, an average *n* value as large as 0.922 is found, which corresponds to relaxation kinetics trends barely distinguishable from the simple exponential course. By contrast, all oxidized samples show significantly lower average *n* values (ranging from 0.771 for sample C to 0.835 for sample B5), which is perfectly consistent with the idea that chemical modification causes surface inhomogeneity. However, no clear trend among the oxidized samples was observed, suggesting complex interactions between treatment conditions and resulting surface properties.

**Figure 5:**
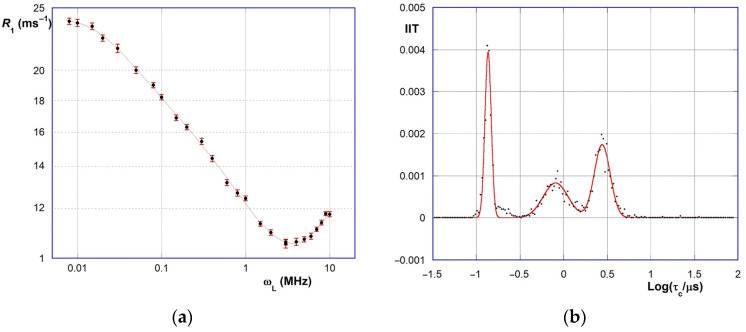
Relaxometric data for material B7: (**a**) NMRD curve; (**b**) IIT curve (Equation (3)) from Monte Carlo analysis.

On analyzing the relaxometric NMRD curves of biochars, a difficulty arises from the possible occurrence of paramagnetic effects, which is indeed typical for graphitic carbonaceous materials [48,49,50,51,52]. In the case of biochars, the origin of this effect is unclear because it might be partly attributed to the possible trace presence of paramagnetic transition metal ions, and partly to the electron energy level distributions of the extensive C*^sp2^*-conjugated structural moieties. Paramagnetic effects usually result in the occurrence of a minimum in the higher-field section of the NMRD curves (usually located between 6 and 8 MHz referring to ^1^H ν_L_ values). Consequently, the rising final section of the curve must be cut off from the subsequent analysis. The complete relaxometric data for the materials studied in this work are collected in the Supporting Information; a typical example of an NMRD curve is depicted in Figure 5a.

As we mentioned previously, correlation time *τ*_c_ values can be retrieved by suitable mathematical manipulation of the NMRD curves. According to literature, the NMRD plots for complex systems can be suitably subjected to model-free analyses, based on the idea that the system itself may be considered as a continuum of microsystems, each behaving in agreement with the BPP model. Therefore, the normalized distribution function of the microsystems can be retrieved via inverse integral transform analysis of the experimental NMRD curve [45,53], according to Equation (2):

(2) $$R_{1}=R_{0}+\frac{3}{10}\frac{\mu_{0}^{2}\hbar^{2}\gamma_{H}^{4}}{r_{HH}^{6}}\int_{\tau_{c}=0}^{\infty }\left(\frac{\tau_{c}}{1+\tau_{c}^{2}\omega_{L}^{2}}+\frac{{4\tau }_{c}}{1+{4\tau }_{c}^{2}\omega_{L}^{2}}\right)f^{*}\;(\tau_{c})d\tau_{c}$$

where *ω_L_* = 2π·*ν_L_*; *R*_0_ is an offset value (accounting for very fast motions such as hydrogen bond exchange), *r*_HH_ is the average H · H distance and f* (*τ*_c_) is the required distribution function. The latter can be obtained by applying a heuristic analysis method recently proposed by us [46]. Interestingly, the f* (*τ*_c_) distribution actually resolves into a set of delta-like functions; i.e., it is a *τ*_c_-value spectrum. Thus, it is possible to tentatively attribute each *τ*_c_ value to a particular motion mode for the water molecules present. The confidence intervals for *τ*_c_ values can be obtained by applying a suitable Monte Carlo procedure [54], which also enables us to identify possible meaningless artifacts present in the original f* (*τ*_c_) distribution. Finally, from the results of the Monte Carlo analysis, it is possible to build the inverse integral transform (IIT) curves (Equation (3)), which enable the best representation of the *τ*_c_ values and the relevant contributions to the overall *R*_1_ values:

(3) $$IIT\left(\tau_{c}\right)=\underset{\omega_{L}\to 0}{\lim}\;\frac{\partial R_{1}}{\partial \omega_{L}}=\frac{3}{2}\frac{\mu_{0}^{2}\hbar^{2}\gamma_{H}^{4}}{r_{HH}^{6}}\cdot \tau_{c}\cdot f^{*}(\tau_{c})$$

A typical IIT curve is shown for exemplificative purposes in Figure 5b. The *τ*_c_ values for the different materials obtained from the IIT plots are collected in Table 3. In general, the IIT curves feature three main peaks, corresponding to as many *τ*_c_ values (indicated hereinafter as *τ*_1_, *τ*_2_ and *τ*_3_, respectively). Therefore, three main motion modes can be envisaged for the water molecules present in the system, which can be tentatively identified with the movements onto the pore surface or within the pore lumen, and with the pore-surface exchange.

For the pristine PB material, *τ*_c_ values are found as large as 1.25, 7.0 and 18.1 μs. In comparison, oxidative treatment always causes a decrease in *τ*_1_ and *τ*_2_ values; conversely, *τ*_3_ values undergo more complicated variations. As far as the effect of the treatment with the sulfonitric mixture is concerned, all three values show a U-shaped trend with both an increase in the reaction temperature (samples B1, B2, B5, B7) and time (samples B3, B4, B5, B6). The same U-shaped trend is found for the piranha mixture oxidation only for *τ*_1_ values, whereas a bell-shaped trend is found for *τ*_2_ (by contrast, no obvious trend is found for *τ*_3_). In all cases, the turning point of these trends is found for the samples (A3, B5) treated at 60 °C for 30 min. These findings are particularly interesting because they agree with the results of the FTIR and WHC analyses. Hence, oxidative treatment apparently enhances the mobility of the water molecules in the pore network. This is perfectly consistent with the idea that the pore surface is made more hydrophilic by the treatment. In fact, solvation of a highly hydrophobic surface requires extensive clusterization of the water molecules, with a consequent reduction in molecular mobility. Conversely, the insertion of hydrophilic functional groups on the pore surface involves the creation in the surroundings of a highly disordered cybotactic region, which favors molecular mobility. Therefore, *τ*_1_ and *τ*_2_ values might be tentatively related to the on-surface and surface–lumen exchange motions of water molecules. Regarding *τ*_3_ values, their wavering values might be related to in-pore movements, which are more strictly connected to pore size distributions. The latter ones are not bound in a simple way to the treatment conditions, as we explained previously; therefore, their outcome is not straightforward to predict.

### 2.6. Adsorption Tests

**Figure 6:**
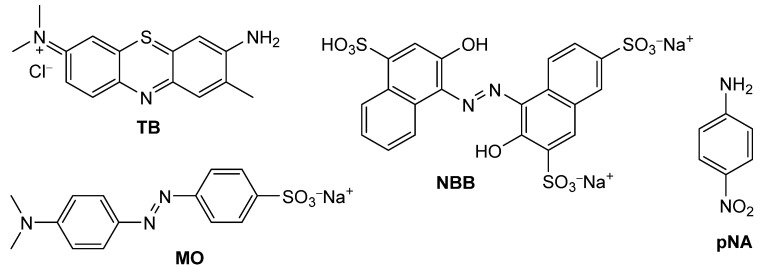
Structures of the dyes used as model pollutants for the adsorption tests.

**Figure 7:**
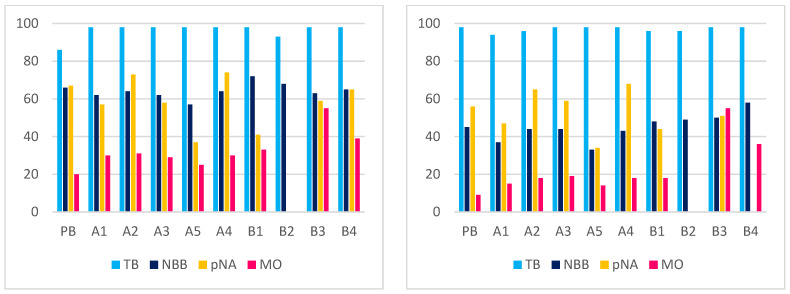
Histograms depicting the adsorption test results at pH 4.4 (**left**) and 6.7 (**right**).

As a final step, the adsorption abilities of the materials were evaluated to assess the potential benefits of functionalization for applications in organic pollutant sequestration. For this purpose, four models of organic dyes—namely Toluidine blue (TB), Naphthol blue black (NBB), 4-nitroaniline (pNA) and methyl orange (MO)—were selected (Figure 6). These dyes were chosen due to their structural diversity and the ease of quantification by UV-Vis spectrophotometry. Adsorption tests were conducted at pH 4.4 and 6.7 to investigate the potential effect of pH on adsorption performance. These values were chosen in analogy with some previous results previously obtained on some nanosponge sorbents [54]. The results are summarized in Table 4 and graphically depicted in Figure 7. The collected data overall indicate a good affinity of the biochar materials for the selected dyes, with some notable exceptions. Biochars treated with nitric acid, or those treated with the sulfonitric mixture under the harshest reaction conditions (samples B5, B6, B7 and C) caused the aqueous dye solutions to acquire a brownish tint upon equilibration. This coloration suggests superficial exfoliation of the biochar, resulting in the release of soluble components. Notably, when equilibrated with TB or NBB, the UV-Vis spectra of the supernatants showed a bathochromic shift (~35 nm) for the residual dye absorption band. This indicates that a fraction of the dye interacts with the exfoliated components of the biochar material through π-π interactions. Hence, these materials were not further investigated.

The results reported in Table 4 enable us to draw further considerations on the overall effect of the oxidizing treatment. In comparison to pristine PB, modification involves a fair improvement in the adsorption abilities on average. In almost half of the cases (33 entries out of 68), percent adsorption increases (up to 6 times in the case of MO with B3 at pH 6.7); however, it must also be mentioned that, in some cases, a decrease (16 entries) or a negligible effect (21 entries) can also be observed. Adsorption is usually more effective at pH 4.4, with few exceptions (5 cases out of 34). By averaging the results for each material, it was determined that the use of the sulfonitric mixture affords the best enhancement in comparison to the piranha mixture. The largest average enhancement is found with material B3. Notably, with both oxidizing systems, the adsorption performances of the materials worsen as the oxidation conditions are harshened. In fact, a significant decrease in the average performances can be observed when comparing A5 to A4, or B4 to B3. Once again, the idea is confirmed that optimizing the material features requires a suitable compromise among the reaction conditions. The reported data also show that the effect of BP pristine functionalization on adsorption varies significantly depending on the type of dye considered. For instance, the cationic TB was almost completely adsorbed by all modified biochars at both pH levels (with BP pristine showing 86% adsorption at pH 4.4), preventing meaningful comparisons between the materials. The dianionic NBB was less efficiently adsorbed than TB but showed better affinity at pH 6.7. In this case, functionalization generally did not result in significant changes in adsorption capacity, except for materials A1 and A5 (which performed worse), and for material B4 as well. Conversely, for pNA, the oxidation of the material does not afford satisfactory results, with few exceptions (A2, A3, A4 and B4 at pH 6.7). Furthermore, MO provides an interesting case of study, because its affinity for pristine PB is only modest. Therefore, in this case, oxidative modification affords the most apparent adsorption improvements, and, in general, allows for the appreciation of the occurrence of optimum oxidation conditions for tuning the features of the materials.

Some final consideration should be made to address the effect of pH variations. The average effect from the acidity of the aqueous medium largely depends on both the nature of the organic model species and the sorbent material. The adsorption of anionic NBB and MO significantly benefits from a pH decrease. This might be tentatively justified assuming that the material is able to bind some hydrogen ions from the solution due to its oxygenated functional groups, assuming a slight positive surface charge density. Conversely, pH sensitivity is low for cationic TB (except for PB). For neutral pNA, pH effects largely vary as the sorbent material changes. For instance, better adsorption is achieved at acidic pH with PB, A1, A2 and B3, whereas the opposite occurs with B4. However, on average, nearly all materials fairly benefit from more acidic conditions, with few exceptions.

## 3. Materials and Methods

### 3.1. Materials and Instrumentation

All the needed commercial reagents and solvents were used as purchased (Merck-Sigma-Aldrich, Darmstdt, Germany) with no further purification. Pristine poplar biochar (PB) was produced in a gassificator. The poplar biomass was heated at 1200 °C for 6 h and subsequently cooled down to room temperature under an ordinary atmosphere.

ATR-FTIR spectra of the various synthesized materials were recorded on a Bruker LUMOS microscope apparatus (Bruker Ltd., Billerica, MA, USA). RAMAN spectra were recorded on a Horiba LabRAM HR Evolution apparatus (Horiba Ltd., Kyoto, Japan) using a 532 nm laser source. UV-Vis spectra were recorded on a Beckmann-Coulter DU 800 spectrometer (Beckman Coulter Inc., Brea, CA, USA). FFC-NMR experiments were carried out on a STELAR SmarTracer 0.25 Tesla Bench-top NMR Relaxometer instrument (Stelar s.r.l., Mede, Italy). Porosimetric experiments were performed on a Quantachrome Nova 2200 Multi-Station High Speed Gas Sorption Analyser (Quantachrome GmBK & Co., Odelzhausen, Germany). SEM images were acquired with an environmental scanning electron microscope, Zeiss EP EVO 50 (Carl Zeiss AG, Oberkochen, Germany), equipped with different secondary electron detectors, usable according to the working pressure range (high vacuum ~10–4 Pa; variable pressure 10–1000 Pa; environmental pressure <3000 Pa), and backscattered electron detectors. It is not necessary to coat non-conductive samples, thanks to the possibility of working at variable pressures. The large chamber allows the insertion of samples with a diameter <250 mm and height <120 mm. The analysis was performed at a variable pressure of 90 Pa. The ChatGPT AI tool (GPT-4o mini) was used for language improvement and revision.

### 3.2. Oxidative Functionalization of PB

#### 3.2.1. Procedure with Piranha Mixture (Products A1–A5)

Conc. H_2_SO_4_ (98%, 3 mL) and 30% aq. H_2_O_2_ (1 mL) were cautiously mixed at 0 °C (ice bath). Then, pristine PB (1.0 g) was added, and the system was placed in an oil bath at the proper temperature for the required reaction time (see Table 1 in the Section 2) under magnetic stirring. The resulting reaction crude was quickly poured into ice-cold water (20 mL); part of the excess acid was neutralized with NaOH (4.0 g, cautiously added in small portions), and then neutralization was completed by slowly adding solid NaHCO_3_ (litmus paper assay). The solid product was allowed to settle overnight, filtered off, repeatedly washed with water, then with methanol, and finally dried in vacuo over P_2_O_5_ at 60 °C overnight.

#### 3.2.2. Procedure with Sulfonitric Mixture (Products B1–B7)

The sulfonitric mixture was first prepared by cautiously mixing at 0 °C (ice bath) H_2_SO_4_ (98%, 3 mL) and conc. (68% aq.) HNO_3_ (1 mL). Then, pristine PB (1.0 g) was added, and the system was placed in an oil bath at the proper temperature for the proper reaction time (see the Section 2) under magnetic stirring. The resulting reaction crude was quickly poured into ice-cold water (20 mL); then, neutralization (partly with NaOH, and completed with NaHCO_3_) and isolation of the product were carried out as described above.

#### 3.2.3. Procedure with Nitric Acid (Product C)

To the pristine PB (1.0 g), placed into a flask equipped with a condenser, 68% conc. HNO_3_ (4 mL) was added, and the mixture was kept in an oil bath at 60 °C for 30 min under magnetic stirring. The resulting reaction crude was quickly poured into ice-cold water (20 mL); then, neutralization (partly with NaOH, and completed with NaHCO_3_) and isolation of the product were carried out as described above.

### 3.3. Percent Water-Holding Capacity (WHC)

A weighed sample of each material (ca. 150–200 mg) was introduced in a 2 mL plastic syringe (after having placed a tiny piece of cotton wool into the needle adapter to avoid any loss of material). The syringe was then placed overnight in a small beaker containing some distilled water, caring to adjust the top of the carbonaceous material at the same level as the liquid (in such a way as to ensure water adsorption only by capillarity). After equilibration, the syringe was weighted, and the WHC was calculated as WHC = (P_f_ − P_i_)/P_i_, where P_f_ and P_i_ are the final and the initial weights of the sample, respectively.

### 3.4. Porosimetry

The N_2_ adsorption and desorption isotherms were recorded at 77 K after the samples were degassed at 200 °C for 2 h in the degas station, in order to remove any moisture and other gases adsorbed onto the surface. The specific surface area was calculated according to the standard BET method. The pore size distributions were calculated using the BJH method applied to the desorption branch of the isotherms.

### 3.5. FFC-NMR Relaxometry

For the FFC-NMR experiments, the materials (ca. 300 mg) were placed in a 9 mm diameter NMR tube and allowed to equilibrate overnight with distilled water. The excess water was drained out just before the experiment. The longitudinal relaxation rates (*R*_1_) were evaluated in the ^1^H Larmor frequency range between 10.0 and 0.01 MHz. Relaxometric data were analyzed using the software packages UpenWin and GUIModelFreeFFC, kindly made available by Prof. Villiam Bortolotti (University of Bologna, Italy). In particular, the latter one was employed to analyze the NMRD dispersion curves (*R*_1_ vs. ν_L_). For this purpose, the integration interval (generally between 0.001 and 10 s; in a few cases 0.005~50 s or 0.01~100 s) was divided into 200 steps; for subsequent Monte Carlo analysis, 401 simulation runs were performed.

### 3.6. Adsorption Tests

Stock solutions (5 × 10^−5^ M) of the model organic pollutants were prepared in aqueous buffer solutions at pH 4.4 and 6.7, prepared in turn as follows. (i) pH 4.4: CH_3_COONa (200 mg) was dissolved in 250 mL of freshly double-distilled water, and then the proper amount of HCl 1 M (7 mL) was added to reach the desired pH value; (ii) pH 6.7: NaH_2_PO_4_ (232 mg) and Na_2_HPO_4_ (113 mg) were dissolved in 250 mL of freshly double-distilled water. The pH of the stock buffer solutions was checked by a CRISON MicropH 2001 instrument (Crison Instruments, Alella, Barcelona, Spain) equipped with a commercial glass electrode and possibly adjusted to the desired value by small additions of standard aq. HCl 1 M or NaOH 1 M.

Samples for the adsorption tests were prepared by adding 2.0 mL of guest solution (at the initial concentration *c_i_* of 5 × 10^−5^ M) to a carefully weighted amount (4.00 ± 0.05 mg) of the sorbent materials. The samples were shaken at r. t. for 90 min in an orbital shaker, and then centrifuged for 15 min at 4000 rpm. The supernatant liquor was carefully pipetted out, and the residual concentration (*c*_f_) of the organic species was determined by UV-Vis spectrophotometry. Finally, the percent adsorption (*%_A_*) was trivially obtained as *%_A_* = (*c*_i_ − *c*_f_)/c_f_.

## 4. Conclusions

The present study demonstrates the successful oxidative functionalization of poplar biochar, using various oxidizing systems, including the piranha mixture (H_2_O_2_/H_2_SO_4_), nitric acid and the sulfonitric mixture (HNO_3_/H_2_SO_4_), under a range of reaction conditions. Special emphasis was placed on evaluating the effects of the reaction temperature and time on the physicochemical properties and performance of the modified biochars. A systematic analysis of the results indicates that achieving a balance between reaction temperature and time is crucial for optimizing the biochar properties. Both the piranha mixture and sulfonitric mixture were most effective at 60 °C for 30 min, conditions that provide the most satisfactory functionalization (in terms of the amount of nitro groups and increase in the WHC value) while minimizing excessive erosion and collapse of the biochar’s porous structure. Moreover, FFC-NMR relaxometry revealed the heterogeneous nature of water dynamics within the oxidized materials. Optimal oxidation conditions resulted in enhanced molecular mobility, supporting the idea that hydrophilic functionalization disrupts water clustering on the surface, improving water accessibility within the pore network. The adsorption tests using model organic dyes demonstrated the potential of oxidative treatments to enhance biochar performance for pollutant sequestration. The sulfonitric mixture generally provided better adsorption performance than the piranha mixture, with material B3 exhibiting the largest improvements. Methyl orange, which showed a limited affinity for pristine biochar, displayed significant adsorption enhancements after oxidative modification, underscoring the ability of functionalization to tune surface properties. The results reinforce the idea that over-oxidation can degrade the biochar structure, reducing its effectiveness for adsorption and water retention. Thus, a compromise between functionalization and structural preservation is essential.

The findings of this work highlight the importance of controlled oxidative functionalization for tuning biochar properties, making it a versatile material for environmental applications such as water purification and soil conditioning. By optimizing reaction conditions, it is possible to enhance biochar’s hydrophilicity, adsorption capacity, and porous network accessibility, which are critical for its practical utility in pollutant sequestration. In summary, this work provides a comprehensive understanding of how oxidative treatments influence biochar’s structural, chemical, and functional properties. It establishes a clear framework for optimizing biochar functionalization to achieve enhanced performance for environmental remediation and resource management applications.

## Figures and Tables

**Table 1:** Summary of the functionalized biochars obtained.

Sample	Oxidant	Temp. (°C)	Time (min)	Yield (*w*/*w*%)	Acidic Groups (meq·g^−1^)
PB	(pristine biochar)	-	-	-	-
A1	Piranha mixture	0	30	87	-
A2	Piranha mixture	27	30	81	-
A3	Piranha mixture	60	30	78	0.64 ± 0.03
A4	Piranha mixture	60	180	86	-
A5	Piranha mixture	90	30	76	0.60 ± 0.03
B1	Sulfonitric mixture	0	30	91	0.32 ± 0.03
B2	Sulfonitric mixture	27	30	90	0.58 ± 0.05
B3	Sulfonitric mixture	60	10	82	0.57 ± 0.02
B4	Sulfonitric mixture	60	30	87	0.51 ± 0.05
B5	Sulfonitric mixture	60	60	83	1.07 ± 0.05
B6	Sulfonitric mixture	60	90	92	1.59 ± 0.05
B7	Sulfonitric mixture	90	30	82	0.77 ± 0.02
C	HNO_3_	60	30	91	-

**Table 2:** WHC values for the functionalized biochars.

Sample	WHC	Sample	WHC	Sample	WHC
PB	2.56 ± 0.07	A5	3.96 ± 0.07	B5	2.81 ± 0.04
A1	3.30 ± 0.08	B1	2.97 ± 0.07	B6	3.10 ± 0.02
A2	3.20 ± 0.14	B2	3.10 ± 0.03	B7	2.79 ± 0.03
A3	3.28 ± 0.04	B3	3.40 ± 0.08	C	3.29 ± 0.02
A4	3.05 ± 0.03	B4	3.29 ± 0.06		

**Table 3:** Correlation time (*τ*_c_) values, from IIT analysis of FFC-NMR data.

Sample	*R*_0_ (ms^−1^)	*τ*_1_ (μs)	*τ*_2_ (μs)	*τ*_3_ (μs)
PB	10.25	1.25 ± 0.03	7.1 ± 0.4	18.3 ± 0.8
A1	9.13	0.63 ± 0.02	2.37 ± 0.13	17.9 ± 0.5
A2	10.20	0.50 ± 0.01	4.18 ± 0.17	16.3 ± 0.8
A3	4.60	0.22 ± 0.01	4.64 ± 0.12	32 ± 2
A4	6.91	0.34 ± 0.01	2.87 ± 0.14	32.3 ± 1.4
A5	7.63	0.45 ± 0.01	2.59 ± 0.1	12.0 ± 0.7
B1	2.97	0.65 ± 0.01	5.9 ± 0.6	19.0 ± 1.2
B2	3.10	0.82 ± 0.02	5.3 ± 0.3	17.8 ± 1.7
B3	3.36	0.33 ± 0.01	1.05 ± 0.06	6.1 ± 0.2
B4	2.10	0.25 ± 0.01	1.02 ± 0.04	4.53 ± 0.4
B5	5.81	0.38 ± 0.01	1.93 ± 0.06	12.7 ± 0.5
B6	5.45	0.41 ± 0.01	2.37 ± 0.09	12.6 ± 0.8
B7	2.79	0.85 ± 0.02	5.1 ± 0.5	17.4 ± 1.1
C	8.73	0.49 ± 0.02	1.55 ± 0.09	7.0 ± 0.2

**Table 4:** Adsorption test data * (percent of guest adsorbed from the starting solution).

Dye	pH	PB	A1	A2	A3	A5	A4	B1	B2	B3	B4
TB	4.4	86	>98	>98	>98	>98	>98	>98	93	>98	>98
	6.7	>98	94	96	>98	>98	>98	96	96	>98	>98
NBB	4.4	66	62	64	62	57	64	72	68	63	65
	6.7	45	37	44	44	33	43	48	49	50	58
pNA	4.4	67	57	73	58	37	74	41	-	59	65
	6.7	56	47	65	59	34	68	44	-	51	>98
MO	4.4	20	30	31	29	25	30	33	-	55	39
	6.7	9	15	18	19	14	18	18	-	55	36

* Average over 3 determinations; all data are given within a ±3% error.

## Data Availability

Dataset available upon request from the authors.

## Supplementary Materials

The following supporting information can be downloaded at https://www.mdpi.com/article/10.3390/molecules30051048/s1: Complete SEM micrographs; FFC-NMR relaxometric data.
