# Supplementary material for: A Study on the Oxidative Functionalization of a Poplar Biochar
Source: Molecules. 2025 Feb 25;30(5):1048. doi: 10.3390/molecules30051048 (PMC11901602; doi:10.3390/molecules30051048)
Supplement: Supplementary file 1 [file molecules-30-01048-s001.zip › molecules-3418871-supplementary.pdf]

A Study on the Oxidative Functionalization of a Poplar Biochar.

A. Di Vincenzo, E. Madonia, C. Librici, P. Bambina, D. Chillura Martino, S. Guernelli, P. Lo Meo, P. Conte

SUPPORTING INFORMATION

Summary

1. Selected SEM micrographs

2. Relaxometric data

1. Selected SEM micrographs

1.1. PB

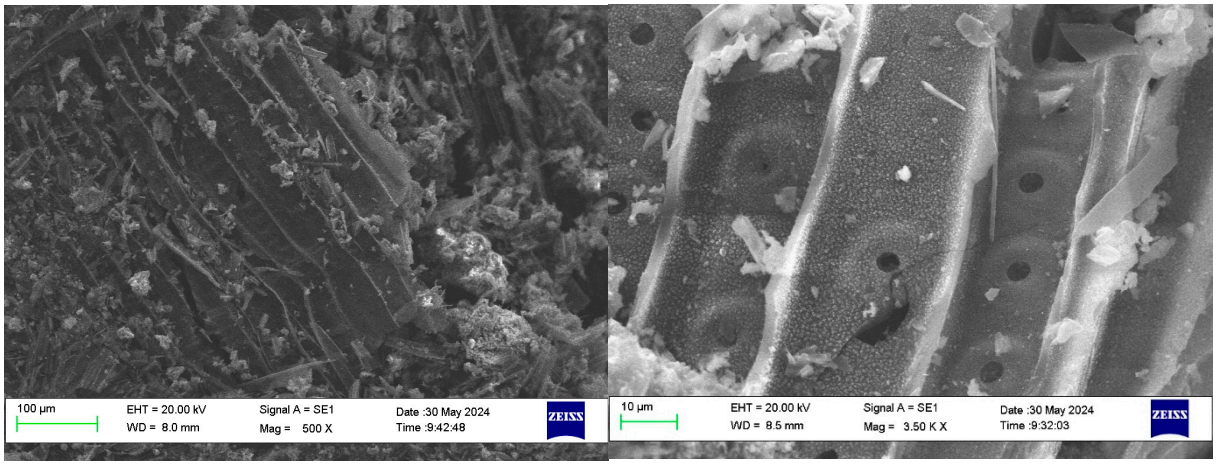

1.2. A2

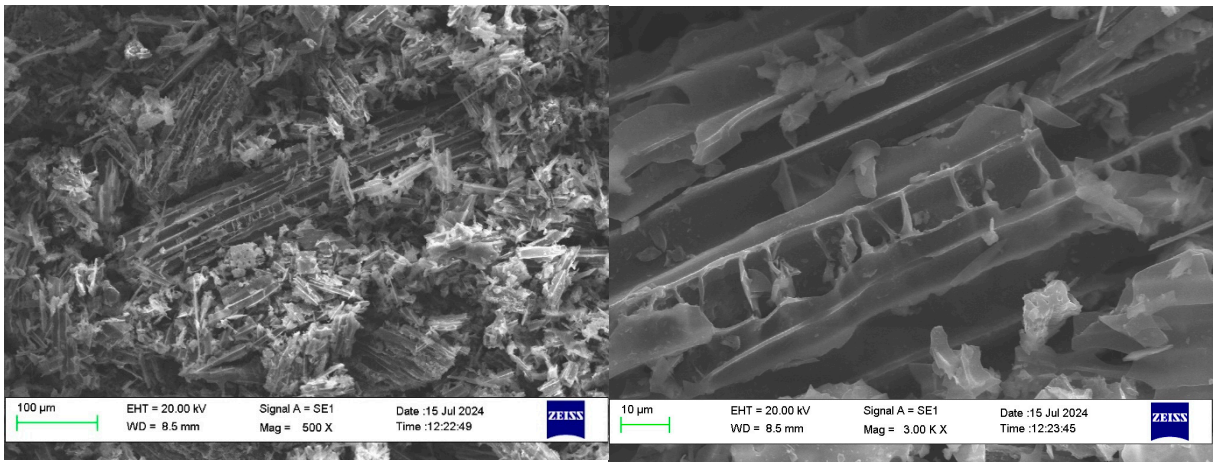

1.3. A3

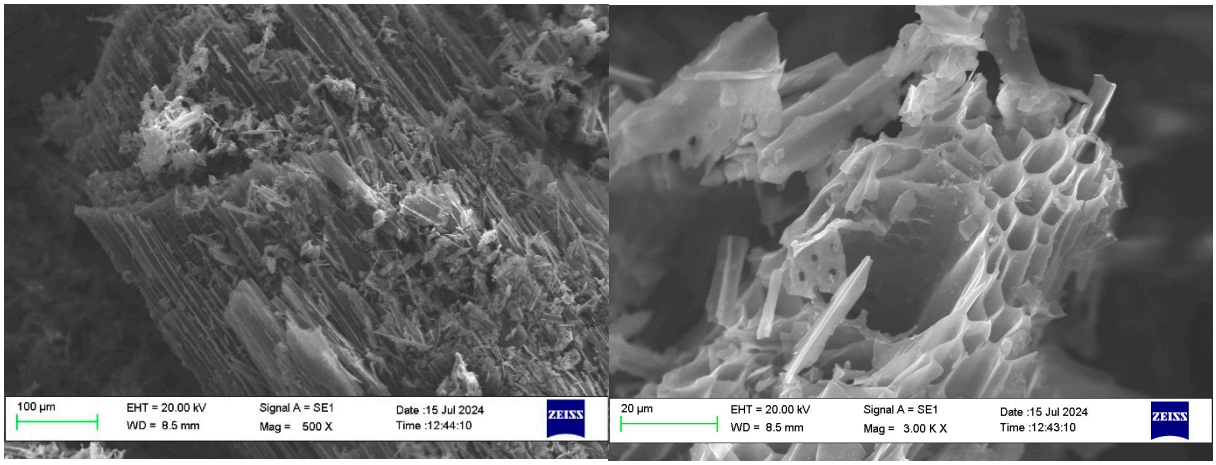

1.4. A4

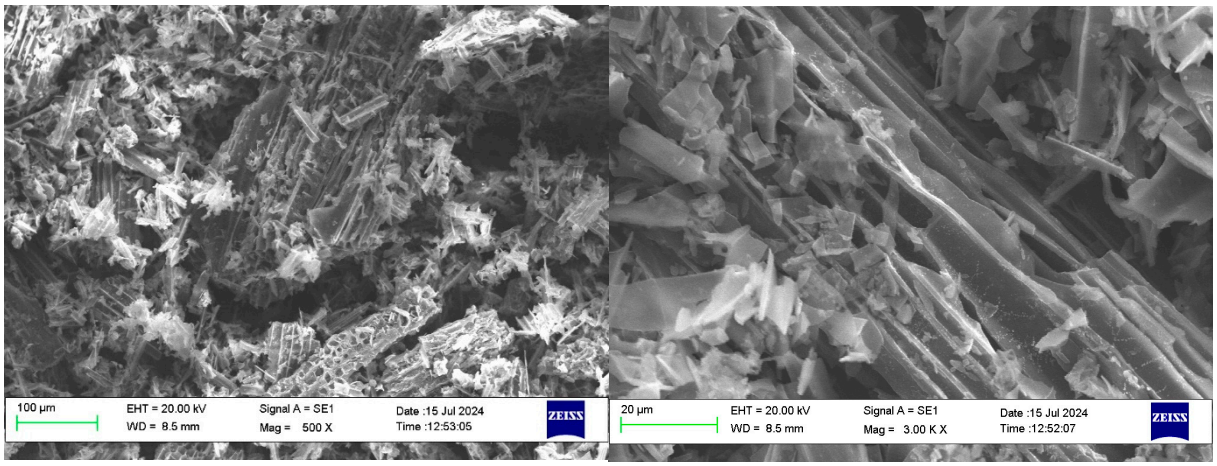

1.5. A5

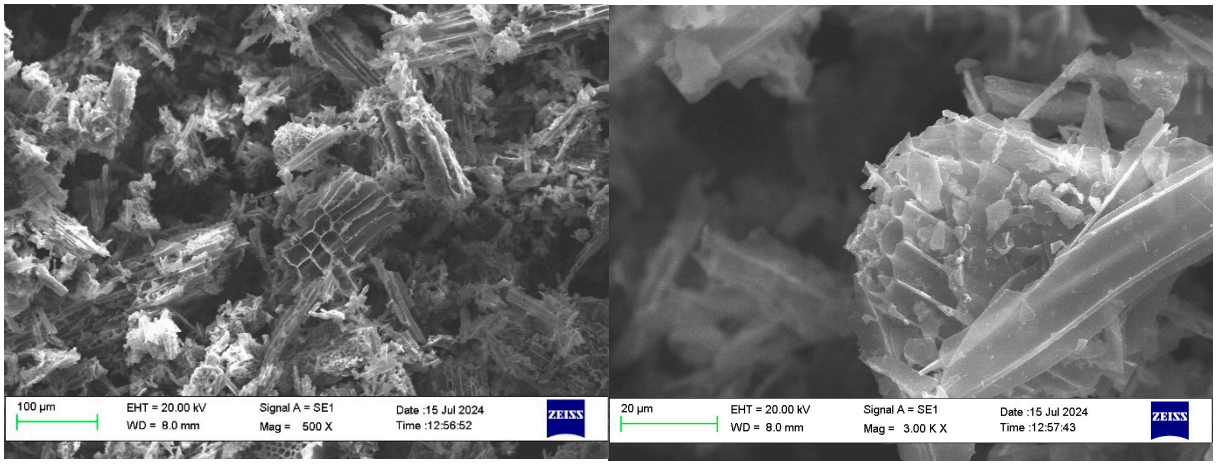

1.6. B1

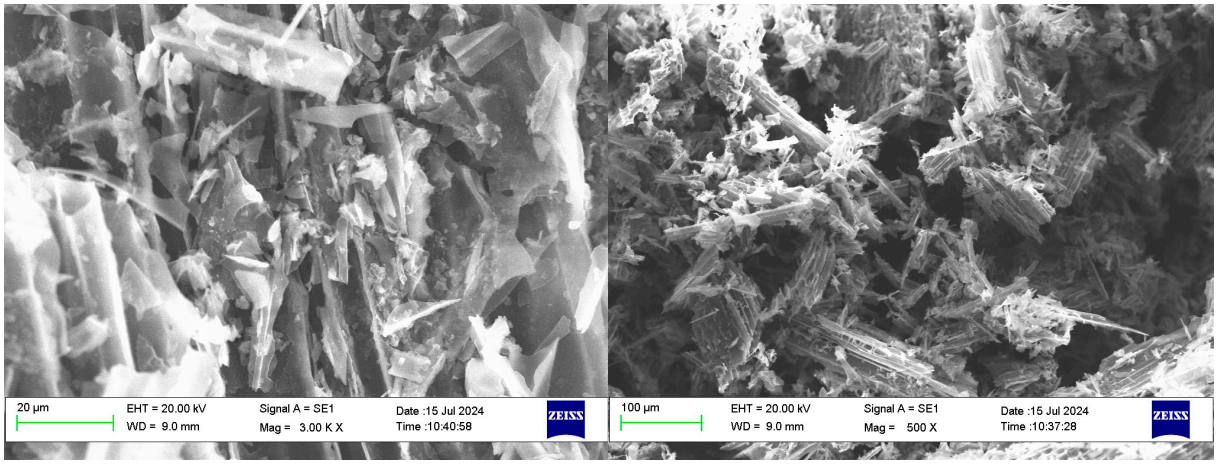

1.7. B2

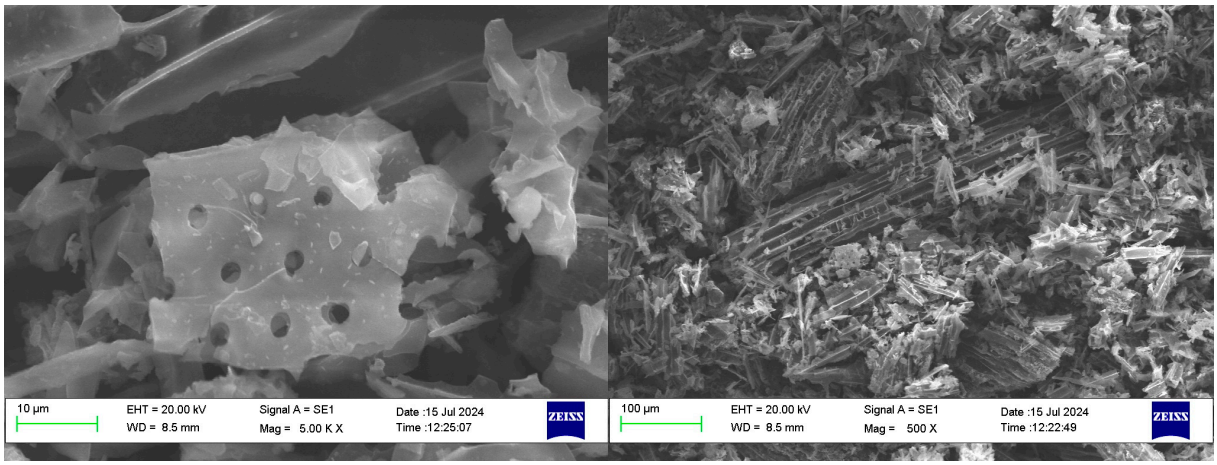

1.8. B3

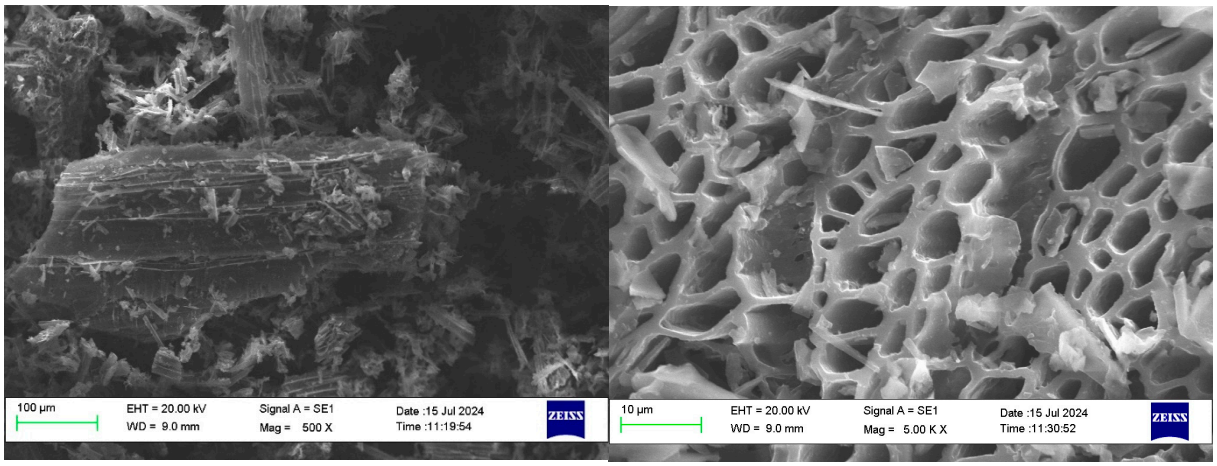

1.9. B4

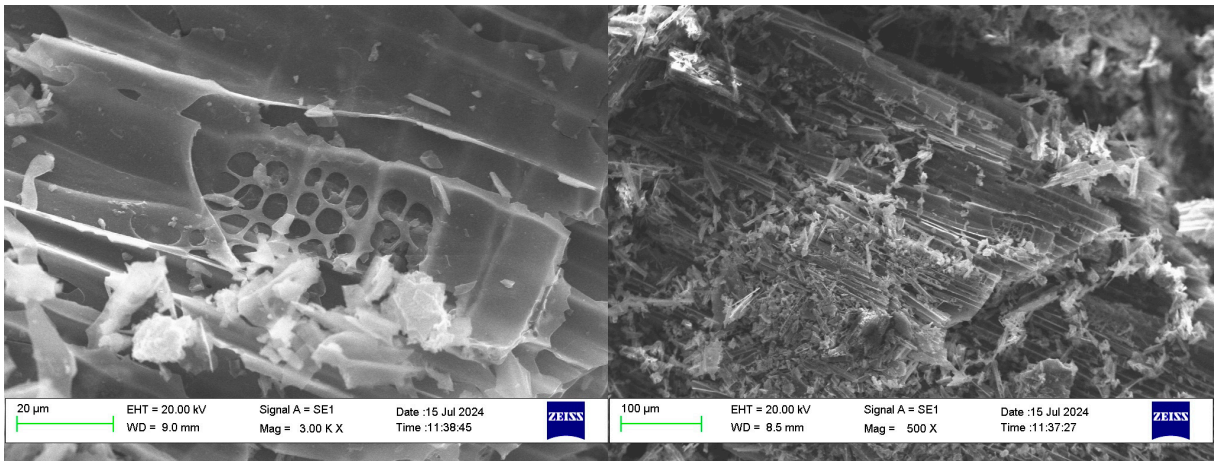

1.10. B5

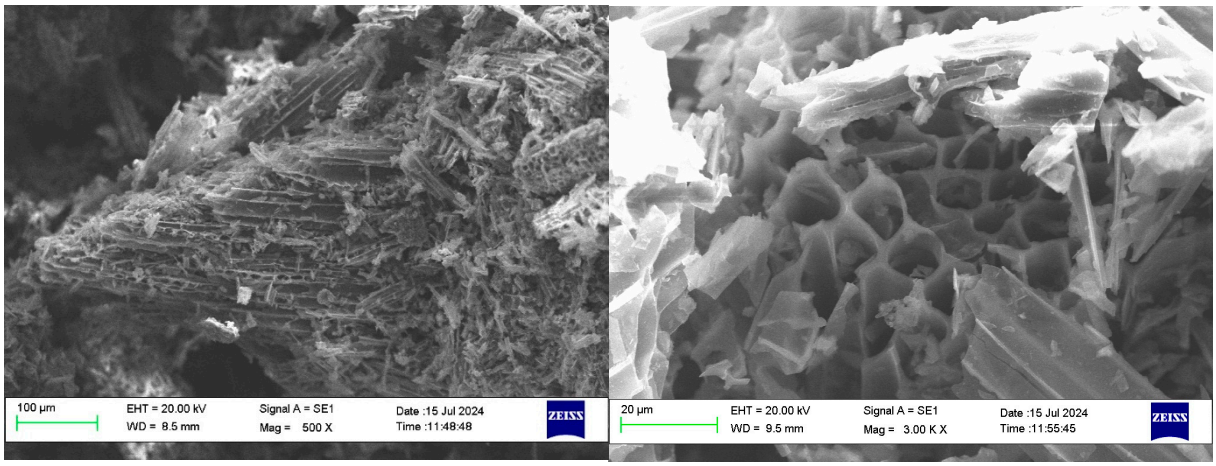

1.11. B6

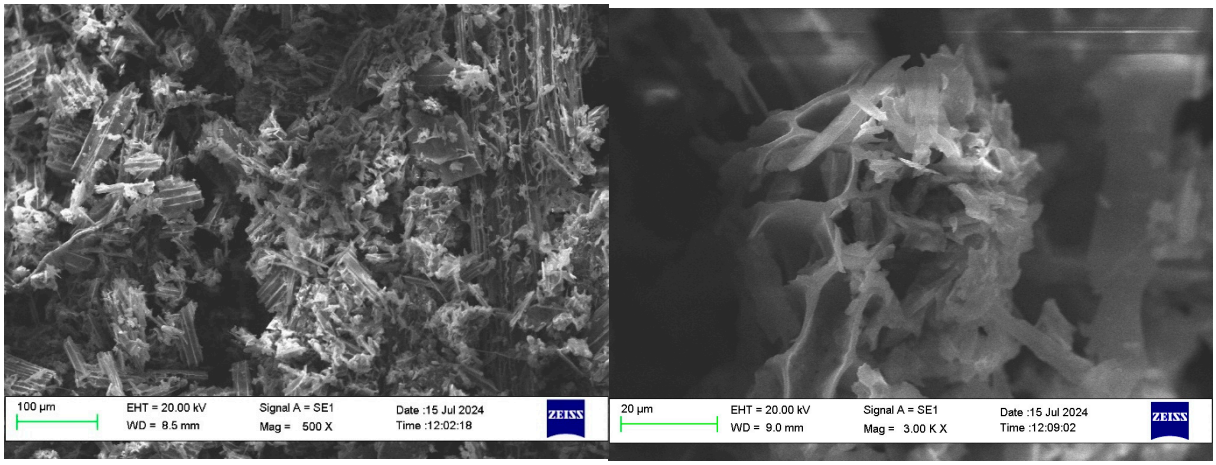

1.12. B7

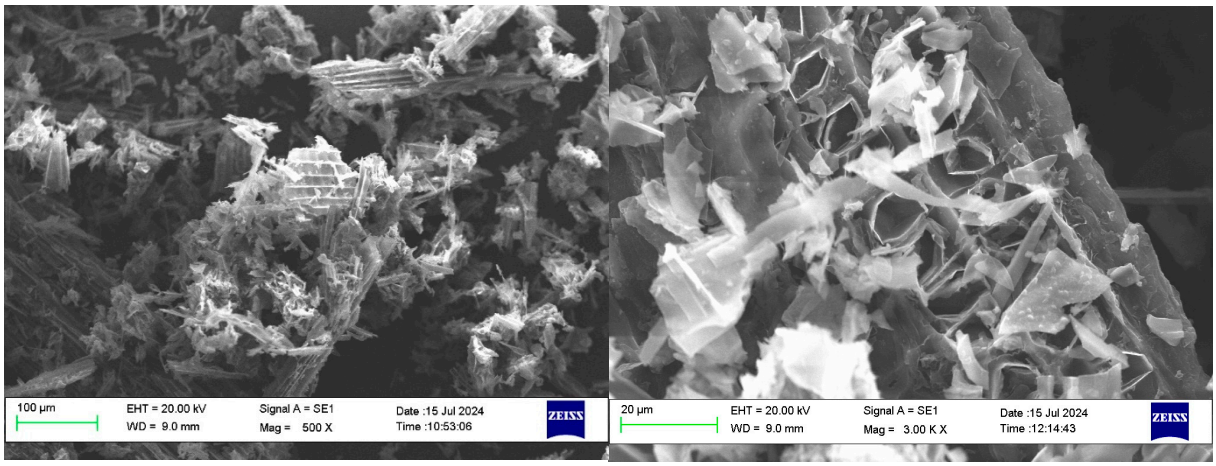

2. Relaxometric data

PB			A1			A2		
ν_L (MHz)	R_1 (ms^{-1})	n	ν_L (MHz)	R_1 (ms^{-1})	n	ν_L (MHz)	R_1 (ms^{-1})	n
10.00	10.34 ± 0.05	0.932	10.00	9.29 ± 0.07	0.790	10.00	10.30 ± 0.10	0.802
9.00	10.43 ± 0.05	0.926	9.00	9.16 ± 0.06	0.789	9.00	10.34 ± 0.06	0.838
8.00	10.36 ± 0.07	0.940	8.00	9.41 ± 0.13	0.806	8.00	10.46 ± 0.11	0.832
7.00	10.30 ± 0.05	0.943	7.00	9.15 ± 0.07	0.788	7.00	10.21 ± 0.09	0.816
6.00	10.23 ± 0.08	0.944	6.00	9.04 ± 0.09	0.789	7.00	10.24 ± 0.07	0.849
5.00	10.23 ± 0.09	0.936	5.00	9.08 ± 0.10	0.787	6.00	10.51 ± 0.10	0.845
4.00	10.48 ± 0.11	0.945	4.00	9.12 ± 0.14	0.773	5.00	10.13 ± 0.08	0.835
3.00	10.48 ± 0.11	0.933	3.00	9.18 ± 0.11	0.793	4.00	10.30 ± 0.13	0.849
3.00	10.26 ± 0.15	0.956	3.00	9.34 ± 0.14	0.770	3.00	10.30 ± 0.14	0.811
2.00	10.84 ± 0.11	0.921	2.00	9.64 ± 0.11	0.779	3.00	10.27 ± 0.17	0.817
1.50	11.20 ± 0.13	0.934	1.50	9.98 ± 0.10	0.772	2.00	10.82 ± 0.15	0.839
1.00	11.70 ± 0.11	0.914	1.00	10.02 ± 0.11	0.779	1.50	11.11 ± 0.14	0.825
0.80	12.22 ± 0.08	0.929	0.80	10.64 ± 0.11	0.776	1.00	11.55 ± 0.15	0.831
0.60	12.65 ± 0.10	0.917	0.60	10.99 ± 0.13	0.767	0.80	11.79 ± 0.13	0.824
0.40	14.03 ± 0.14	0.915	0.40	11.45 ± 0.10	0.779	0.60	12.25 ± 0.13	0.828
0.30	15.15 ± 0.16	0.911	0.30	11.64 ± 0.14	0.772	0.40	12.66 ± 0.13	0.820
0.20	16.25 ± 0.14	0.906	0.20	12.35 ± 0.11	0.763	0.30	13.11 ± 0.10	0.822
0.15	17.06 ± 0.16	0.912	0.15	12.69 ± 0.15	0.775	0.20	13.67 ± 0.13	0.817
0.10	18.52 ± 0.20	0.904	0.10	13.08 ± 0.13	0.772	0.15	13.84 ± 0.16	0.831
0.08	19.84 ± 0.19	0.920	0.08	13.29 ± 0.15	0.763	0.10	14.37 ± 0.12	0.820
0.05	21.54 ± 0.21	0.915	0.05	13.92 ± 0.14	0.774	0.08	15.06 ± 0.13	0.827
0.03	23.54 ± 0.23	0.912	0.03	14.46 ± 0.16	0.759	0.05	15.69 ± 0.16	0.800
0.02	25.09 ± 0.26	0.899	0.02	15.29 ± 0.14	0.752	0.030	15.94 ± 0.18	0.814
0.015	26.13 ± 0.28	0.913	0.015	15.55 ± 0.19	0.765	0.020	16.86 ± 0.18	0.836
0.010	26.56 ± 0.27	0.903	0.010	15.80 ± 0.18	0.781	0.015	17.10 ± 0.18	0.836
0.008	26.97 ± 0.31	0.885	0.008	15.82 ± 0.15	0.768	0.010	17.99 ± 0.23	0.828
						0.008	17.94 ± 0.14	0.818

A3			A4			A5		
ν_L (MHz)	R_1 (ms^{-1})	n	ν_L (MHz)	R_1 (ms^{-1})	n	ν_L (MHz)	R_1 (ms^{-1})	n
10.00	4.24 ± 0.03	0.824	10.00	6.98 ± 0.06	0.790	10.00	7.75 ± 0.04	0.790
9.00	4.54 ± 0.04	0.807	9.00	6.92 ± 0.05	0.821	9.00	7.74 ± 0.05	0.804
8.00	4.31 ± 0.04	0.822	8.00	6.99 ± 0.05	0.821	8.00	7.69 ± 0.07	0.803
6.00	4.36 ± 0.05	0.810	7.00	7.09 ± 0.06	0.815	7.00	7.74 ± 0.04	0.811
4.00	4.52 ± 0.06	0.824	5.00	7.12 ± 0.08	0.808	6.00	7.68 ± 0.06	0.804
3.10	5.52 ± 0.05	0.844	4.00	7.18 ± 0.10	0.804	5.00	7.67 ± 0.08	0.814
3.00	5.33 ± 0.08	0.815	6.00	6.96 ± 0.07	0.816	4.00	7.75 ± 0.10	0.823
2.00	5.82 ± 0.07	0.836	3.00	7.05 ± 0.13	0.793	3.00	7.82 ± 0.12	0.792
1.50	6.29 ± 0.10	0.832	3.00	7.54 ± 0.09	0.819	3.00	7.93 ± 0.10	0.804
1.00	6.66 ± 0.08	0.830	2.00	7.86 ± 0.10	0.813	2.00	7.95 ± 0.10	0.816
0.80	6.84 ± 0.10	0.825	1.50	8.11 ± 0.10	0.792	1.50	8.46 ± 0.09	0.813
0.60	7.52 ± 0.09	0.832	1.00	8.35 ± 0.11	0.799	1.00	8.69 ± 0.10	0.811
0.40	7.80 ± 0.08	0.818	0.80	8.63 ± 0.11	0.796	0.80	8.92 ± 0.08	0.796
0.40	7.71 ± 0.10	0.810	0.60	8.70 ± 0.11	0.798	0.60	9.26 ± 0.09	0.784
0.30	7.98 ± 0.11	0.802	0.40	9.09 ± 0.11	0.805	0.40	9.26 ± 0.09	0.784
0.20	8.51 ± 0.11	0.809	0.30	9.27 ± 0.10	0.795	0.20	10.40 ± 0.08	0.788
0.15	8.77 ± 0.10	0.795	0.20	9.74 ± 0.15	0.806	0.15	10.76 ± 0.10	0.778
0.10	9.63 ± 0.10	0.780	0.15	10.03 ± 0.11	0.806	0.10	11.08 ± 0.11	0.785
0.08	10.15 ± 0.09	0.797	0.10	10.27 ± 0.10	0.797	0.08	11.58 ± 0.10	0.770
0.05	10.41 ± 0.14	0.808	0.08	10.50 ± 0.13	0.803	0.05	12.20 ± 0.12	0.773
0.03	10.96 ± 0.10	0.784	0.05	11.10 ± 0.15	0.790	0.03	12.83 ± 0.11	0.770
0.02	11.52 ± 0.12	0.792	0.03	11.31 ± 0.14	0.793	0.020	13.19 ± 0.15	0.774
0.01	12.00 ± 0.16	0.786	0.020	11.59 ± 0.14	0.786	0.015	13.51 ± 0.11	0.769
			0.015	12.43 ± 0.13	0.813	0.010	13.65 ± 0.15	0.771
			0.010	12.60 ± 0.19	0.813	0.008	13.75 ± 0.14	0.778
			0.008	12.73 ± 0.15	0.827			

B1			B2			B3		
ν_L (MHz)	R_1 (ms^{-1})	n	ν_L (MHz)	R_1 (ms^{-1})	n	ν_L (MHz)	R_1 (ms^{-1})	n
10.00	9.29 ± 0.07	0.790	10.00	10.34 ± 0.06	0.838	10.00	3.40 ± 0.02	0.812
9.00	9.16 ± 0.06	0.789	9.00	10.46 ± 0.11	0.832	9.00	3.44 ± 0.03	0.807
8.00	9.41 ± 0.13	0.806	8.00	10.21 ± 0.09	0.816	8.00	3.41 ± 0.03	0.793
7.00	9.15 ± 0.07	0.788	7.00	10.51 ± 0.10	0.845	7.00	3.49 ± 0.03	0.812
6.00	9.04 ± 0.09	0.789	6.00	10.24 ± 0.07	0.849	6.00	3.48 ± 0.03	0.802
5.00	9.08 ± 0.10	0.787	7.00	10.13 ± 0.08	0.835	5.00	3.58 ± 0.05	0.810
4.00	9.12 ± 0.14	0.773	5.00	10.30 ± 0.13	0.849	4.00	3.62 ± 0.04	0.823
3.00	9.18 ± 0.11	0.793	4.00	10.30 ± 0.14	0.811	3.00	3.65 ± 0.05	0.813
3.00	9.34 ± 0.14	0.770	3.00	10.27 ± 0.17	0.817	3.00	3.88 ± 0.05	0.772
2.00	9.64 ± 0.11	0.779	3.00	10.82 ± 0.15	0.839	2.00	4.06 ± 0.04	0.760
1.50	9.98 ± 0.10	0.772	2.00	11.11 ± 0.14	0.825	1.50	4.26 ± 0.04	0.778
1.00	10.02 ± 0.11	0.779	1.50	11.55 ± 0.15	0.831	1.00	4.75 ± 0.04	0.751
0.80	10.64 ± 0.11	0.776	1.00	11.79 ± 0.13	0.824	0.80	4.79 ± 0.04	0.761
0.60	10.99 ± 0.13	0.767	0.80	12.25 ± 0.13	0.828	0.60	5.00 ± 0.05	0.736
0.40	11.45 ± 0.10	0.779	0.60	12.66 ± 0.13	0.820	0.40	5.49 ± 0.06	0.756
0.30	11.64 ± 0.14	0.772	0.40	13.11 ± 0.10	0.822	0.20	6.00 ± 0.07	0.738
0.20	12.35 ± 0.11	0.763	0.30	13.67 ± 0.13	0.817	0.10	6.67 ± 0.08	0.718
0.15	12.69 ± 0.15	0.775	0.20	13.84 ± 0.16	0.831	0.08	7.24 ± 0.07	0.734
0.10	13.08 ± 0.13	0.772	0.15	14.37 ± 0.12	0.820	0.05	7.44 ± 0.07	0.728
0.08	13.29 ± 0.15	0.763	0.10	15.06 ± 0.13	0.827	0.03	8.05 ± 0.08	0.709
0.05	13.92 ± 0.14	0.774	0.08	15.69 ± 0.16	0.800	0.02	8.22 ± 0.10	0.720
0.03	14.46 ± 0.16	0.759	0.05	15.94 ± 0.18	0.814	0.01	8.72 ± 0.09	0.699
0.02	15.29 ± 0.14	0.752	0.03	16.86 ± 0.18	0.836			
0.015	15.55 ± 0.19	0.765	0.020	17.10 ± 0.18	0.836			
0.010	15.80 ± 0.18	0.781	0.015	17.99 ± 0.23	0.828			
0.008	15.82 ± 0.15	0.768	0.010	17.94 ± 0.14	0.818			
			0.008	17.58 ± 0.20	0.831			

B4			B5			B6		
ν_L (MHz)	R_1 (ms^{-1})	n	ν_L (MHz)	R_1 (ms^{-1})	n	ν_L (MHz)	R_1 (ms^{-1})	n
10.00	2.23 ± 0.02	0.844	10.00	6.00 ± 0.04	0.864	10.00	6.01 ± 0.10	0.820
9.00	2.19 ± 0.02	0.827	9.00	5.91 ± 0.02	0.853	9.00	5.94 ± 0.04	0.815
8.00	2.22 ± 0.02	0.832	8.00	5.96 ± 0.05	0.848	8.00	5.80 ± 0.05	0.790
7.00	2.27 ± 0.02	0.841	7.00	5.96 ± 0.05	0.852	7.00	5.90 ± 0.05	0.783
6.00	2.28 ± 0.02	0.843	6.00	5.97 ± 0.07	0.868	6.00	5.78 ± 0.05	0.783
4.00	2.38 ± 0.03	0.845	5.00	5.90 ± 0.06	0.857	5.00	5.96 ± 0.07	0.780
3.00	2.44 ± 0.04	0.822	4.00	6.10 ± 0.07	0.874	4.00	6.10 ± 0.08	0.792
3.00	2.77 ± 0.05	0.813	3.00	6.15 ± 0.11	0.843	3.00	6.06 ± 0.10	0.774
2.00	2.99 ± 0.03	0.794	3.00	6.60 ± 0.10	0.871	3.00	6.47 ± 0.08	0.804
1.50	3.14 ± 0.04	0.781	2.50	6.65 ± 0.08	0.838	2.00	7.00 ± 0.08	0.787
1.00	3.34 ± 0.04	0.777	2.00	7.01 ± 0.09	0.842	1.50	7.60 ± 0.10	0.788
0.80	3.52 ± 0.04	0.776	1.50	7.17 ± 0.07	0.838	1.25	7.89 ± 0.09	0.786
0.60	3.61 ± 0.04	0.756	1.00	7.83 ± 0.09	0.834	1.00	8.14 ± 0.11	0.780
0.40	4.09 ± 0.05	0.764	0.80	8.25 ± 0.10	0.823	0.80	8.44 ± 0.09	0.787
0.30	4.31 ± 0.07	0.742	0.60	8.88 ± 0.11	0.846	0.60	9.04 ± 0.11	0.772
0.20	4.62 ± 0.07	0.739	0.40	9.48 ± 0.09	0.843	0.40	9.79 ± 0.13	0.774
0.15	4.92 ± 0.06	0.736	0.30	10.18 ± 0.14	0.828	0.30	10.13 ± 0.13	0.765
0.10	5.58 ± 0.08	0.761	0.20	10.94 ± 0.09	0.820	0.20	10.95 ± 0.15	0.758
0.08	5.91 ± 0.08	0.753	0.15	11.79 ± 0.10	0.838	0.15	11.56 ± 0.15	0.753
0.05	6.26 ± 0.08	0.726	0.10	12.31 ± 0.14	0.815	0.10	12.13 ± 0.15	0.742
0.03	6.52 ± 0.08	0.721	0.08	12.99 ± 0.13	0.826	0.08	12.55 ± 0.14	0.755
0.02	6.92 ± 0.10	0.725	0.05	13.73 ± 0.12	0.813	0.05	13.42 ± 0.19	0.744
0.01	7.33 ± 0.10	0.709	0.03	15.10 ± 0.15	0.800	0.03	14.25 ± 0.19	0.731
			0.02	15.79 ± 0.18	0.804	0.020	14.88 ± 0.20	0.729
			0.01	16.52 ± 0.18	0.805	0.015	15.09 ± 0.25	0.716
			0.008	17.13 ± 0.19	0.806	0.010	15.55 ± 0.20	0.714

B7			A1		
ν_L (MHz)	R_1 (ms^{-1})	n	ν_L (MHz)	R_1 (ms^{-1})	n
10.00	11.76 ± 0.12	0.834	10.00	9.38 ± 0.09	0.757
9.00	11.78 ± 0.09	0.839	9.00	9.17 ± 0.10	0.754
8.00	11.40 ± 0.11	0.814	8.00	8.98 ± 0.11	0.770
7.00	11.12 ± 0.10	0.833	7.00	8.93 ± 0.10	0.747
6.00	10.84 ± 0.13	0.824	6.00	8.87 ± 0.16	0.760
5.00	10.72 ± 0.11	0.835	5.00	8.59 ± 0.11	0.764
4.00	10.62 ± 0.15	0.820	4.00	8.58 ± 0.14	0.768
3.00	10.54 ± 0.17	0.816	3.50	8.84 ± 0.22	0.759
3.00	10.61 ± 0.12	0.827	3.00	8.84 ± 0.15	0.757
2.00	10.99 ± 0.12	0.815	2.80	9.72 ± 0.16	0.800
1.50	11.35 ± 0.11	0.821	2.70	9.05 ± 0.13	0.755
1.00	12.46 ± 0.12	0.811	2.50	9.47 ± 0.14	0.780
0.80	12.71 ± 0.16	0.810	2.30	9.29 ± 0.11	0.795
0.60	13.20 ± 0.16	0.800	2.00	9.41 ± 0.11	0.750
0.40	14.43 ± 0.18	0.813	1.50	9.36 ± 0.13	0.753
0.30	15.37 ± 0.19	0.799	1.00	9.82 ± 0.13	0.744
0.20	16.20 ± 0.18	0.795	0.90	10.49 ± 0.10	0.768
0.15	16.76 ± 0.19	0.789	0.80	10.67 ± 0.12	0.784
0.10	18.08 ± 0.19	0.804	0.60	10.91 ± 0.12	0.787
0.08	18.90 ± 0.20	0.798	0.40	11.35 ± 0.14	0.777
0.05	19.97 ± 0.26	0.795	0.30	11.88 ± 0.13	0.758
0.03	21.64 ± 0.36	0.787	0.20	12.69 ± 0.14	0.760
0.020	22.48 ± 0.28	0.801	0.15	12.94 ± 0.13	0.787
0.015	23.47 ± 0.31	0.778	0.10	13.43 ± 0.11	0.771
0.010	23.76 ± 0.34	0.785	0.08	14.20 ± 0.14	0.775
0.008	23.91 ± 0.30	0.790	0.05	15.10 ± 0.16	0.797
			0.03	15.37 ± 0.18	0.806
			0.02	16.09 ± 0.19	0.773
			0.01	16.56 ± 0.17	0.790
